# Relationship between urinary tobacco-specific nitrosamine 4-(methylnitrosamino)-1-(3-pyridyl)-1-butanol (NNAL) and lung function: Evidence from NHANES 2007–2012

**DOI:** 10.18332/tid/175009

**Published:** 2023-12-12

**Authors:** Xiong Lei, Hezhi Wen, Zhixiao Xu

**Affiliations:** 1 Department of Pulmonary and Critical Care Medicine, The First Affiliated Hospital of Wenzhou Medical University, Wenzhou, China

**Keywords:** tobacco, NNAL, lung function, spirometry, NHANES

## Abstract

**INTRODUCTION:**

4-(methylnitrosamino)-1-(3-pyridyl)-1-butanol (NNAL), a metabolite of tobacco-specific nitrosamine (TSNA) 4-(methylnitrosamino)-1-(3-pyridyl)-1-butanone (NNK), is a tobacco-specific carcinogen. Spirometry values (FEV1%, PEF%, etc.) are commonly used as clinical indicators to assess the condition of lung function and the results can be used to diagnose respiratory diseases. However, the relationship between urinary NNAL levels and lung function is unclear.

**METHODS:**

We performed a secondary dataset analysis of the three cycles of the National Health and Nutrition Examination Survey (NHANES) from 2007 to 2012. The association of urinary NNAL with spirometry values was assessed using weighted linear models. In addition, subgroup analyses by gender were also tested.

**RESULTS:**

One unit increased in urinary NNAL could result in a 28% decrease of FEV1/FVC% (mean difference, MD= -0.28; 95% CI: -0.39 – -0.17), 44% decrease of FEV1% (MD= -0.44; 95% CI: -0.69 – -0.18), and FEV1/FEV6% and FEV3/FEV6% decreased by 20% and 8%, respectively. Increased urinary NNAL was associated with lower PEF% (MD= -0.85; 95% CI: -1.19 – -0.51), FEF25-75% (MD= -1.40; 95% CI: -1.94 – -0.87), and FENO (MD= -0.67; 95% CI: -0.92 – -0.42). But forced expiratory time (FET) showed an increment (MD=0.10; 95% CI: 0.03–0.16). The FEV1/FEV6% and FEV3/FEV6% showed decreasing trend from the lowest urinary NNAL quartiles to the highest urinary NNAL quartiles, while FET showed an increased trend. PEF%, FEF 25–75%, and FENO showed the same decreasing trend (all p<0.05). In addition, urinary NNAL seemed to affect spirometry values more in males.

**CONCLUSIONS:**

Urinary NNAL was negatively correlated with FEV1/FVC%, FEV1%, FEV1/FEV6%, FEV3/FEV6%, PEF%, FEF25–75%, and FENO, which was closely related to lung function.

## INTRODUCTION

Cigarette smoke is reported to contain 72 carcinogens^1^. Among the various carcinogens found in tobacco and tobacco smoke, tobacco-specific nitrosamines (TSNAs) are an important class of carcinogens agents. 4-(methylnitrosamino)-1-(3-pyridyl)-1-butanol (NNAL), a metabolite of TSNAs, is present in urine^2^. 4-(methylnitrosamino)-1-(3-pyridyl)-1-butanone (NNK) is a semi-volatile TSNAs, and exposure to NNK is primarily assessed by its urinary metabolite, with urinary NNAL being a good predictor of total NNK intake^3,4^. In a study that included 179 children, it was reported that >96% were found to have detectable urinary NNAL^5^.

In recent years, an increasing number of studies have focused on the relationship between urinary NNAL level and disease. A study found a negative correlation between tobacco-specific urinary NNAL and cognitive function^6^. In children and adolescents, individuals with higher levels of NNAL in their urine were 1.614 times more likely to have a developmental disorder (DDs) compared to their counterparts who were exposed to environmental tobacco smoke^7^. Another study showed higher urinary NNAL levels increased the risk of children attending an urgent medical appointment within 6 months^8^.

As a metabolite of smoking, NNAL level is also associated with respiratory disease. A prospective community-based cohort study revealed the risk of developing lung cancer with increasing levels of urinary NNAL^9^. NNAL exposure was found to be associated with enhanced migration and chemoresistance in lung cancer cells by a mechanism due to NNAL-induced phosphorylation of liver kinase B1 (LKB1) and its loss of function^10^. It was observed that smokers exhibited significantly elevated urinary NNAL levels compared to non-smokers, a phenomenon that might be closely associated with an increased susceptibility to chronic obstructive pulmonary disease (COPD)^11^.

Assessment of spirometry values is helpful in assessing respiratory and cardiovascular health. A recent study proposed that low-dose CT screening in lung cancer combined with spirometry could help identify patients with undiagnosed early COPD^12^. A prospective cohort study demonstrated that the risk of atrial fibrillation increases linearly with decreasing ratio of forced expiratory volume in one second to forced vital capacity (FEV1/FVC%)^13^. But it is not clear exactly which lung functions urinary NNAL affects.

In our study, we aimed to assess the associations between urinary NNAL and lung function in adults participating in National Health and Nutrition Examination Survey (NHANES). Attempts were made to reveal the basis of the physiological effects that cause respiratory diseases.

## METHODS

### Study participants

Three survey cycles of NHANES data (2007–2012) were included by us to perform a secondary dataset analysis in this study. NHANES is a major program of the National Centre for Health Statistics (NCHS) of the USA that provides the nation with vital health statistics. The program conducts a series of surveys that focus on different population groups or health topics.

**Figure 1:**
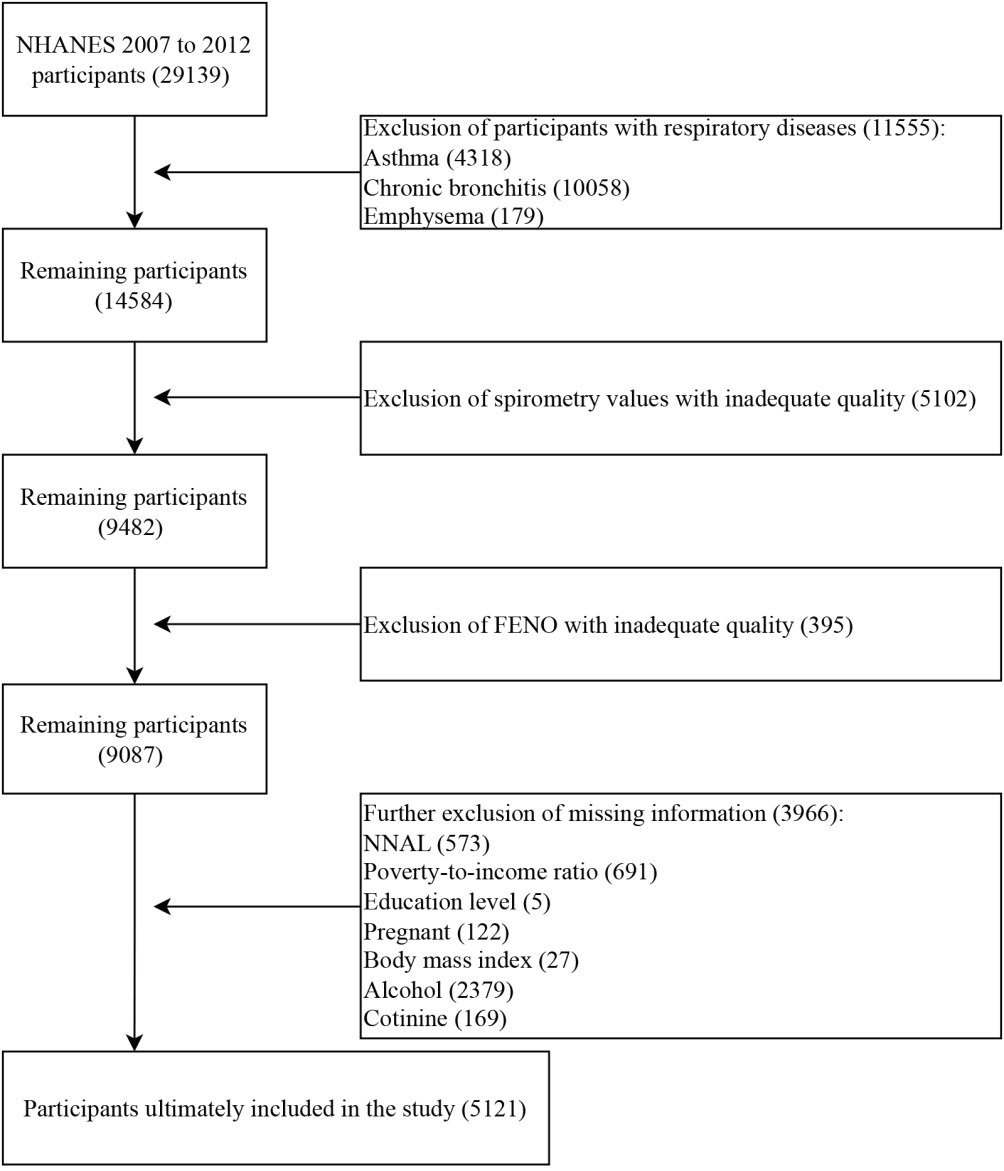
Flowchart of participant selection

In NHANES 2007–2012, participants had complete spirometry values and urinary NNAL data at the same time, therefore a total of 29139 participants from the three survey cycles were included in our study. First, we excluded 11555 participants with respiratory diseases, including asthma (4318), chronic bronchitis (10085), and emphysema (179). Next, we excluded 5102 participants with inadequate spirometry data, and 395 participants with inadequate fractional exhaled nitric oxide (FENO) data. Then, we excluded 3966 participants who were missing other information, including 573 participants who were missing NNAL information, 691 participants who were missing poverty-to-income ratio (PIR) information, 5 participants who were missing education level, 27 participants who were missing body mass index (BMI) data, 2379 participants who were missing alcohol information, and 169 participants who were missing cotinine information. Finally, a total of 5121 participants were included in our study (Figure 1).

### Exposure definitions

The nitrosamine NNK is an important component of tobacco and tobacco smoke. In smokers, NNK is rapidly reduced to its metabolite NNAL, and a large fraction of NNAL may also exist in the form of glucuronide NNAL-Gluc (NNAL-N-Gluc and NNAL-O-Gluc). NNAL can be used as a biomarker of exposure to NNK in active smokers, as well as in non-smokers, after exposure to secondhand smoke (SHS). Our study used total urinary NNAL, and for the ‘total’ NNAL assay, NNAL was measured using liquid chromatography coupled to tandem mass spectrometry (LC/MS/MS). The NNAL concentration was derived from the ratio of the integral peaks of the natural ions to the labeled ions by comparing it to a standard calibration curve.

We corrected urinary NNAL by creatinine as in a previous study^14^, and NNAL-creatinine correction (NNAL-CR) was generated as NNAL μg/g of creatinine, and then we converted the results to log10-transformed; and then obtained quartiles and the three cutoff positions. Each interval had a corresponding number of participants.

### Outcomes

Spirometry values were included in our study outcome. The spirometry procedure is followed the recommendations of the American Thoracic Society (ATS). All spirometer data collected were reviewed by expert consultants from the National Institute for Occupational Safety and Health (NIOSH) quality control center. Spirometry with a grade quality of A and B was accepted for our study. The spirometers were from Ohio and maintained by the NIOSH laboratory. To minimize the risk of infection for participants, spirometry hoses were fitted with a new in-line biological filter (A-M Systems PFT Filter Kit B). In the process of measuring spirometry values, participants were asked to blow out air for at least 6 s and were able to fulfill the following three requirements: a reproducible graph of spirometry was obtained, up to 8 spirometry curves could be obtained, and until the participant was unable to continue blowing air. In our study, the spirometry values included forced vital capacity in the predicted (FVC%), forced expiratory volume in 1 s predicted (FEV1%), FEV1/FVC%, forced expiratory volume in the 1 s/forced expiratory volume in the first 6 s ratio (FEV1/FEV6%), forced expiratory volume in the 3 s/forced expiratory volume in the first 6 s ratio (FEV3/FEV6%), peak expiratory flow in predicted (PEF%), forced expiratory flow between 25–75% in predicted (FEF25–75%), and forced expiratory time (FET). In addition, FENO was included in our outcomes. FENO was measured by the Aerocrine NIOX MINO, a portable and hand-held NO analyzer. The FENO value was the average of two repeatable measurements. The calculations of the predicted values of the relevant spirometry indicators for each of the ethnic groups were derived from this study^15^.

### Covariates

The covariates included age, gender, race/ethnicity, education level, PIR, BMI, alcohol, and cotinine. The race/ethnicity was categorized into five categories: Mexican American, non-Hispanic White, non-Hispanic Black, and Other (including multi-racial). Education level was divided into three categories: less than high school, high school/general educational development, and above high school. Cotinine, as a metabolite of nicotine, is an indicator that can present in both active and passive smoking participants. It is more reliable than smoking and non-smoking reported in questionnaire form. Urinary NNAL and cotinine are closely related, but NNAL has a longer half-life than cotinine. Cotinine is a short-time indicator of smoking and used to measure recent tobacco smoke exposure, therefore cotinine was included in our covariates.

### Data analysis

The weighting scheme recommended by the official NHANES website was used to analyze the data. All continuous variables were tested for normal distribution. Median and interquartile range (IQR), and unweighted frequencies (weighted percentages) were used to indicate non-normal continuous, and categorical variables, respectively. The chi-squared and Kruskal-Wallis tests were used to test categorical and continuous variables, respectively, to compare the level of characteristic differences between NNAL (quartiles) groups.

We assessed the correlation between NNAL and the continuous outcomes using weighted linear models. Lowest quartile group was used as the reference group in order to analyze the relationship between NNAL and study outcomes among the four groups. We also considered quartiles as continuous variables and performed a test for trend (p for trend). In addition, subgroup analyses according to gender was performed and p for interaction was tested. Model 1 was adjusted by age, gender. Model 2 was adjusted by age, gender, race/ethnicity, education level, and PIR. And Model 3 was adjusted by age, gender, race/ethnicity, education level, PIR, BMI, alcohol, and cotinine. A p<0.05 was considered significant, and all data analyses were performed at R4.2.2.

## RESULTS

### Baseline characteristics

The baseline characteristics of the population recruited to the study are shown in Table 1. A total of 5121 participants were recruited, including 2902 males and 2219 females. The population was predominantly Non-Hispanic (72.9%), highest urinary NNAL level populations were more likely to be the Non-Hispanic White group. Participants in the highest urinary NNAL quartile tended to have lower PIR, BMI, FEV1/FVC%, FEV1/FEV6%, FEV3/FEV6%, PEF, PEF%, FEF25–27, FEF25–75%, and FENO compared to those in the lowest NNAL quartile. FVC was higher in the fourth urinary NNAL quartile when compared with the first urinary NNAL quartile. The cotinine and alcohol were also higher in the highest NNAL quartile. However, there were no significant differences in FVC%.

**Table 1:** Baseline characteristics of participants by quartiles of the urinary NNAL, NHANES, 2007–2012

	*ALL n (%)*	*Q1 (≤0.6419) n (%)*	*Q2 (0.6419–0.4308) n (%)*	*Q3 (0.4308–3.5902) n (%)*	*Q4 (>3.5902) n (%)*	*p*
	*(N=5121)*	*(N=1256)*	*(N=1222)*	*(N=1278)*	*(N=1365)*	
**Age** (years), median (IQR)	43 (31–54)	47 (36–58)	44 (32–57)	39 (28–52)	41 (30–51)	<0.001
**Gender**						<0.001
Male	2902 (54.4)	747 (59.7)	572 (43.3)	696 (50.5)	887 (64.1)	
Female	2219 (45.6)	509 (40.3)	650 (56.7)	582 (49.5)	478 (35.9)	
**Race/ethnicity**						<0.001
Mexican American	845 (8.3)	262 (9.5)	217 (8.5)	215 (9.0)	151 (6.1)	
Other Hispanic	515 (4.9)	141 (4.8)	142 (5.6)	129 (5.4)	103 (3.9)	
Non-Hispanic White	2457 (72.9)	567 (72.6)	586 (74.3)	595 (71.6)	709 (73.0)	
Non-Hispanic Black	911 (8.5)	184 (6.7)	156 (5.6)	242 (9.2)	329 (12.4)	
Other	393 (5.4)	102 (6.4)	121 (6.0)	97 (4.8)	73 (4.5)	
**Education level**						<0.001
<High school	1003 (12.8)	172 (7.0)	170 (8.3)	260 (12.8)	401 (23.0)	
>High school	3036 (67.7)	879 (78.3)	854 (78.0)	737 (66.0)	566 (48.4)	
High school /general educational development	1082 (19.6)	205 (14.7)	198 (13.7)	281 (21.3)	398 (28.6)	
	***median (IQR)***	***median (IQR)***	***median (IQR)***	***median (IQR)***	***median (IQR)***	***p***
PIR	3.60 (1.88–5.00)	4.62 (2.72–5.00)	4.17 (2.31–5.00)	3.36 (1.65–5.00)	2.49 (1.17–3.43)	<0.001
BMI (kg/m^2^)	27.30 (23.95–31.30)	27.70 (24.65–31.80)	27.15 (23.70–30.93)	27.85 (24.00–31.90)	26.80 (23.20–30.88)	<0.001
Cotinine (ng/mL)	0.04 (0.01–19.11)	0.01 (0.01–0.03)	0.02 (0.01–0.03)	0.13 (0.03–0.71)	205.00 (109.69–304.80)	<0.001
Alcohol (drinks)	2 (1–3)	2 (1–3)	2 (1–2)	2 (1–4)	3 (2–5)	<0.001
FVC (mL)	4253 (3567–5057)	4357 (3636–5075)	3999 (3450–4746)	4177 (3511–5068)	4458 (3751–5286)	<0.001
FEV1 (mL)	3351 (2798–3981)	3419 (2849–4000)	3191 (2733–3819)	3349 (2793–4026)	3443 (2858–4054)	<0.001
FVC%	100 (92–108)	99 (92–108)	100 (91–108)	100 (92–108)	100 (92–108)	0.908
FEV1%	98 (90–106)	99 (90–107)	99 (92–107)	99 (91–106)	100 (92–108)	<0.001
FEV1/FVC%	79 (75–83)	79 (75–83)	80 (76–84)	80 (76–84)	78 (72–82)	<0.001
FEV6 (mL)	4121 (3457–4891)	4231 (3509–4904)	3886 (3355–4627)	4101 (3409–4926)	4299 (3615–5107)	<0.001
FEV3 (mL)	3939 (3294–4682)	4026 (3328–4698)	3745 (3192–4455)	3915 (3266–4736)	4116 (3450–4867)	<0.001
FEV1/FEV6%	81 (78–85)	81 (78–84)	82 (79–85)	82 (79–85)	80 (76–84)	<0.001
FEV3/FEV6%	96 (94–97)	95 (94–97)	96 (94–97)	96 (94–97)	95 (93–97)	<0.001
PEF (mL)	8605 (7265–10317)	9000 (7436–10644)	8257 (7147–10047)	8605 (7241–10291)	8603 (7224–10206)	<0.001
PEF%	106 (95–117)	109 (98–119)	108 (99–119)	107 (96–118)	101 (89–112)	<0.001
FEF25–75 (mL)	3135 (2269–4012)	3099 (2256–3940)	3157 (2351–3922)	3297 (2385–4245)	3010 (2113–3942)	<0.001
FEF25–75%	92 (74–111)	93 (72–112)	97 (79–114)	85 (78–114)	86 (63–102)	<0.001
FET (s)	10.50 (8.60–12.70)	11.00 (9.00–13.00)	10.10 (8.30–12.10)	10.10 (8.40–12.04)	11.00 (8.80–13.90)	<0.001
FENO (ppb)	13 (9–20)	16 (11–22)	14 (10–20)	14 (10–20)	8 (6–13)	<0.001

PIR: poverty-to-income ratio. BMI: body mass index. FVC: forced vital capacity. FEV1: forced expiratory volume in 1 s. FVC%: forced vital capacity in the predicted. FEV1%: forced expiratory volume in 1 s predicted. FEV1/FVC%: forced expiratory volume in the first 1 s/forced vital capacity predicted ratio. FEV6: forced expiratory volume in the first 6 s. FEV3: forced expiratory volume in the first 3 s. FEV1/FEV6%: forced expiratory volume in 1 s/forced expiratory volume in the first 6 s ratio. FEV3/FEV6%: forced expiratory volume in 3 s/forced expiratory volume in the first 6 s ratio. PEF: peak expiratory flow rate. PEF%: peak expiratory flow in predicted. FEF25–75: forced expiratory flow between 25%–75%. FEF25–75%: forced expiratory flow between 25%–75% in predicted. FET: forced expiratory time. FENO: fractional exhaled nitric oxide. Q1: the lowest NNAL group. Q2: the lower NNAL group. Q3: the higher NNAL group. Q4: the highest NNAL group. IQR: interquartile range.

### Association between the NNAL and study outcomes

Table 2 demonstrated the relationship between urinary NNAL and spirometry values. In all the models we explored, we did not find a relationship between urinary NNAL and FVC%. We found strong associations in the rest of the indicators. In Model 3, one-unit increase in urinary NNAL resulted in a decrease in FEV1/FVC% (mean difference, MD= -0.28; 95% CI: -0.39 – -0.17) and FEV1% (MD= -0.44; 95% CI: -0.69 – -0.18). Urinary NNAL increased by one unit, FEV1/FEV6% and FEV3/FEV6% decreased by 20% and 8%, respectively. Increased urinary NNAL was associated with lower PEF% (MD= -0.85; 95% CI: -1.19 – -0.51), FEF25–75% (MD= -1.40; 95% CI: -1.94 – -0.87), and FENO (MD= -0.67; 95% CI: -0.92 – -0.42). However, FET showed an increment (MD=0.10; 95% CI: 0.03–0.16).

**Table 2:** Association of urinary NNAL with study outcomes, NHANES, 2007–2012

*Spirometry*	*Model 1 MD (95% CI)*	*p*	*Model 2 MD (95% CI)*	*p*	*Model 3 MD (95% CI)*	*p*
FEV1/FVC%	-0.50 (-0.60 – -0.39)	<0.001	-0.49 (-0.60 – -0.39)	<0.001	-0.28 (-0.39 – -0.17)	<0.001
FEV1%	-0.68 (-0.86 – -0.50)	<0.001	-0.64 (-0.82 – -0.47)	<0.001	-0.44 (-0.69– -0.18)	0.001
FVC%	-0.06 (-0.22–0.10)	0.438	-0.03 (-0.20–0.14)	0.689	-0.08 (-0.30–0.14)	0.478
FEV1/FEV6%	-0.37 (-0.46 – -0.29)	<0.001	-0.37 (-0.46 – -0.29)	<0.001	-0.20 (-0.29 – -0.11)	<0.001
FEV3/FEV6%	-0.16 (-0.19 – -0.13)	<0.001	-0.15 (-0.18 – -0.12)	<0.001	-0.08 (-0.11 – -0.05)	<0.001
PEF%	-1.17 (-1.39 – -0.94)	<0.001	-1.02 (-1.25 – -0.79)	<0.001	-0.85 (-1.19 – -0.51)	<0.001
FEF25–75%	-2.09 (-2.52 – -1.66)	<0.001	-2.08 (-2.53 – -1.63)	<0.001	-1.40 (-1.94 – -0.87)	<0.001
FET (s)	0.15 (0.10–0.19)	<0.001	0.15 (0.11–0.20)	<0.001	0.10 (0.03–0.16)	0.004
FENO (ppb)	-1.17 (-1.34 – -0.99)	<0.001	-1.18 (-1.35 – -1.01)	<0.001	-0.67 (-0.92 – -0.42)	<0.001

MD: mean difference. FEV1/FVC%: forced expiratory volume in the 1 s/forced vital capacity predicted ratio. FEV1%: forced expiratory volume in the 1 s predicted. FVC%: forced vital capacity in the predicted. FEV1/FEV6%: forced expiratory volume in the 1 s/forced expiratory volume in the first 6 s ratio. FEV3/FEV6%: forced expiratory volume in the 3 s/forced expiratory volume in the first 6 s ratio. PEF%: peak expiratory flow in predicted. FEF25–75%: forced expiratory flow between 25%–75% in predicted. FET: forced expiratory time. FENO: fractional exhaled nitric oxide. Model 1 was adjusted for age and gender. Model 2 was adjusted for age, gender, race/ethnicity, education level, and poverty-to-income ratio. Model 3 was adjusted for age, gender, race/ethnicity, education level, poverty-to-income ratio, body mass index, alcohol, and cotinine.

### Association of quartiles of NNAL on study outcomes

The relationship between spirometry values and urinary NNAL quartiles is shown in Table 3. In Model 3, the higher the urinary NNAL, the lower the FEV1/FVC% (p for trend=0.002). However, there was no significant relationship between urinary NNAL quartiles and FEV1% or FEV%. The FEV1/FEV6% and FEV3/FEV6% also showed decreasing trend from the lowest urinary NNAL quartiles to the highest urinary NNAL quartiles (all p for trend <0.05). In Model 3, PEF%, FEF25-75%, and FENO showed the same decreasing trend (all p<0.05). FET showed an increased trend from the lowest urinary NNAL quartiles (MD= -0.29; 95% CI: -0.56 – -0.01) to the highest NNAL quartiles (MD=0.89; 95% CI: 0.28–1.50).

**Table 3:** Effect of urinary NNAL quartiles on spirometry values, NHANES, 2007– 2012

*Spirometry*	*Q1*	*Q2*		*Q3*		*Q4*	
		*MD (95% CI)*	*p*	*MD (95% CI)*	*p*	*MD (95% CI)*	*p*
**FEV1/FVC%**							
Model 1	Ref.	0.37 (-0.25–0.98)	0.234	-0.07 (-0.56–0.43)	0.790	-2.96 (-3.69 – -2.22)	<0.001
Model 2	Ref.	0.40 (-0.21–1.00)	0.190	-0.06 (-0.57–0.46)	0.823	-2.89 (-3.57 – -2.20)	<0.001
Model 3	Ref.	0.44 (-0.15–1.04)	0.138	-0.04 (-0.56–0.48)	0.889	-1.54 (-2.30 – -0.78)	<0.001
**FEV1%**							
Model 1	Ref.	-0.02 (-1.33–1.30)	0.979	-0.78 (-2.06 – 0.50)	0.227	-4.01 (-5.35 – -2.66)	<0.001
Model 2	Ref.	0.05 (-1.28–1.38)	0.938	-0.67 (-1.98–0.64)	0.307	-3.67 (-5.03 – -2.31)	<0.001
Model 3	Ref.	-0.04 (-1.38–1.30)	0.955	-0.66 (-1.96–0.63)	0.305	-1.86 (-3.79–0.07)	0.059
**FVC%**							
Model 1	Ref.	-0.49 (-1.91–0.94)	0.493	-0.68 (-1.95–0.59)	0.287	-0.28 (-1.54–0.97)	0.651
Model 2	Ref.	-0.43 (-1.87–1.02)	0.554	-0.60 (-1.90–0.71)	0.362	-0.07 (-1.41–1.26)	0.914
Model 3	Ref.	-0.58 (-1.99–0.83)	0.410	-0.61 (-1.90–0.67)	0.339	0.14 (-1.59–1.86)	0.874
**FEV1/FEV6%**							
Model 1	Ref.	0.29 (-0.24–0.81)	0.278	0.01 (-0.41–0.43)	0.966	-2.23 (-2.82 – -1.63)	<0.001
Model 2	Ref.	0.31 (-0.21–0.83)	0.239	-0.004 (-0.45–0.44)	0.987	-2.21 (-2.77 – -1.64)	<0.001
Model 3	Ref.	0.36 (-0.15–0.87)	0.163	0.01 (-0.44–0.45)	0.973	-1.09 (-1.75 – -0.44)	0.002
**FEV3/FEV6%**							
Model 1	Ref.	0.16 (-0.02–0.34)	0.087	-0.05 (-0.20–0.10)	0.469	-0.92 (-1.15 – -0.69)	<0.001
Model 2	Ref.	0.17 (-0.02–0.35)	0.074	-0.04 (-0.19–0.11)	0.613	-0.87 (-1.09 – -0.66)	<0.001
Model 3	Ref.	0.16 (-0.03–0.34)	0.095	-0.02 (-0.17–0.13)	0.750	-0.41 (-0.63 – -0.18)	0.001
**PEF%**							
Model 1	Ref.	-0.61 (-2.16–0.95)	0.434	-1.86 (-3.63 – -0.10)	0.039	-7.69 (-9.46 – -5.92)	<0.001
Model 2	Ref.	-0.43 (-1.97–1.11)	0.575	-1.41 (-3.15–0.33)	0.109	-6.64 (-8.37 – -4.91)	<0.001
Model 3	Ref.	-0.39 (-1.94–1.15)	0.608	-1.39 (-3.15–0.37)	0.118	-5.23 (-7.53 – -2.93)	<0.001
**FEF25–75%**							
Model 1	Ref.	1.93 (-0.77–4.63)	0.156	-0.84 (-3.40–1.72)	0.512	-12.18 (-15.09 – -9.27)	<0.001
Model 2	Ref.	1.93 (-0.76–4.62)	0.154	-0.86 (-3.42–1.70)	0.500	-11.93 (-14.86 – -8.99)	<0.001
Model 3	Ref.	2.14 (-0.51–4.80)	0.110	-0.84 (-3.44–1.76)	0.515	-6.88 (-10.54 – -3.22)	0.001
**FET** (s)							
Model 1	Ref.	-0.24 (-0.52–0.03)	0.081	0.05 (-0.32–0.43)	0.786	1.10 (0.72–1.49)	<0.001
Model 2	Ref.	-0.27 (-0.55–0.004)	0.053	0.08 (-0.32–0.48)	0.704	1.20 (0.77–1.63)	<0.001
Model 3	Ref.	-0.29 (-0.56 – -0.01)	0.045	0.06 (-0.34–0.46)	0.766	0.89 (0.28–1.50)	0.005
**FENO** (ppb)							
Model 1	Ref.	-1.42 (-2.82 – -0.01)	0.048	-0.29 (-1.89–1.30)	0.714	-8.26 (-9.82 – -6.69)	<0.001
Model 2	Ref.	-1.39 (-2.80–0.01)	0.052	-0.25 (-1.82–1.31)	0.747	-8.25 (-9.77 – -6.74)	<0.001
Model 3	Ref.	-1.39 (-2.81–0.03)	0.054	-0.13 (-1.73–1.47)	0.869	-5.88 (-7.71 – -4.06)	<0.001

MD: mean difference. FEV1/FVC%: forced expiratory volume in the 1 s/forced vital capacity predicted ratio. FEV1%: forced expiratory volume in the 1 s predicted. FVC%: forced vital capacity in the predicted. FEV1/FEV6%: forced expiratory volume in the 1 s/forced expiratory volume in the first 6 s ratio. FEV3/FEV6%: forced expiratory volume in the 3 s/forced expiratory volume in the first 6 s ratio. PEF%: peak expiratory flow in predicted. FEF25–75%: forced expiratory flow between 25–75% in predicted. FET: forced expiratory time. FENO: fractional exhaled nitric oxide. Model 1 was adjusted for age and gender. Model 2 was adjusted for age, gender, race/ethnicity, education level, and poverty-to-income ratio. Model 3 was adjusted for age, gender, race/ethnicity, education level, poverty-to-income ratio, body mass index, alcohol, and cotinine.

### Effect modification of NNAL on study outcomes by gender

Table 4 showed the relationship of urinary NNAL with study outcomes by gender. Overall, the urinary NNAL seemed to affect spirometry values more in males. There appeared to be no gender differences in the effect of urinary NNAL on FVC%, with p>0.05 for all models. However, gender differences were shown in the remaining spirometry values (FEV1/FVC%, FEV1%, FEV1/FEV6%, FEV3/FEV6%, FEF25-75%, PEF%, FET, and FENO) in relationship to urinary NNAL (all p<0.05).

**Table 4:** Consequence modification of urinary NNAL on spirometry values by gender, NHANES, 2007–2012

*Spirometry*	*Model 1*	*p*	*p for interaction*	*Model 2*	*p*	*p for interaction*	*Model 3*	*p*	*p for interaction*
	*MD (95% CI)*			*MD (95% CI)*			*MD (95% CI)*		
FEV1/FVC%									
Male	-0.49 (-0.65 – -0.33)	<0.001	<0.001	-0.46 (-0.62 – -0.31)	<0.001	<0.001	-0.36 (-0.52 – -0.21)	<0.001	0.001
Female	-0.51 (-0.64 – -0.39)	<0.001		-0.54 (-0.69 – -0.38)	<0.001		-0.10 (-0.29–0.08)	0.251	
FEV1%									
Male	-0.59 (-0.85 – -0.33)	<0.001	<0.001	-0.56 (-0.81 – -0.31)	<0.001	<0.001	-0.56 (-0.85 – -0.26)	<0.001	0.019
Female	-0.82 (-1.05 – -0.59)	<0.001		-0.79 (-1.05 – -0.53)	<0.001		-0.18 (-0.62–0.26)	0.410	
FVC%									
Male	0.04 (-0.22–0.29)	0.779	0.902	0.04 (-0.22–0.29)	0.757	0.815	-0.08 (-0.38–0.21)	0.566	0.882
Female	-0.21 (-0.43–0.02)	0.068		-0.17 (-0.42–0.08)	0.182		-0.06 (-0.45–0.33)	0.741	
FEV1/FEV6%									
Male	-0.36 (-0.50 – -0.23)	<0.001	<0.001	-0.34 (-0.47 – -0.21)	<0.001	<0.001	-0.26 (-0.38 – -0.13)	<0.001	0.005
Female	-0.39 (-0.49 – -0.29)	<0.001		-0.42 (-0.53 – -0.30)	<0.001		-0.06 (-0.21–0.10)	0.463	
**FEV3/FEV6%**									
Male	-0.14 (-0.19 – -0.10)	<0.001	<0.001	-0.13 (-0.18 – -0.09)	<0.001	<0.001	-0.09 (-0.13 – -0.05)	<0.001	0.006
Female	-0.18 (-0.22 – -0.14)	<0.001		-0.18 (-0.23 – -0.14)	<0.001		-0.05 (-0.11–0.01)	0.09	
**PEF%**									
Male	-1.09 (-1.40 – -0.79)	<0.001	<0.001	-0.96 (-1.26 – -0.67)	<0.001	<0.001	-1.12 (-1.52 – -0.72)	<0.001	<0.001
Female	-1.28 (-1.61 – -0.95)	<0.001		-1.12 (-1.51 – -0.74)	<0.001		-0.33 (-0.92–0.27)	0.276	
**FEF25-75%**									
Male	-1.95 (-2.55 – -1.34)	<0.001	<0.001	-1.90 (-2.53 – -1.27)	<0.001	<0.001	-1.82 (-2.49 – -1.15)	<0.001	<0.001
Female	-2.31 (-2.91 – -1.70)	<0.001		-2.37 (-3.09 – -1.64)	<0.001		-0.52 (-1.50–0.46)	0.292	
**FET** (s)									
Male	0.16 (0.10–0.21)	<0.001	<0.001	0.16 (0.11–0.22)	<0.001	<0.001	0.13 (0.05–0.21)	0.001	0.023
Female	0.13 (0.07–0.20)	<0.001		0.13 (0.06–0.21)	0.001		0.03 (-0.05–0.11)	0.423	
**FENO** (ppb)									
Male	-1.24 (-1.49 – -0.99)	<0.001	<0.001	-1.21 (-1.44 – -0.99)	<0.001	<0.001	-0.65 (-1.02 – -0.28)	0.001	<0.001
Female	-1.05 (-1.23 – -0.87)	<0.001		-1.13 (-1.36 – -0.89)	<0.001		-0.73 (-1.11– -0.36)	<0.001	

MD: mean difference. FEV1/FVC%: forced expiratory volume in the 1 s/forced vital capacity predicted ratio. FEV1%: forced expiratory volume in the 1 s predicted. FVC%: forced vital capacity in the predicted. FEV1/FEV6%: forced expiratory volume in the 1 s/forced expiratory volume in the first 6 s ratio. FEV3/FEV6%: forced expiratory volume in the 3 s/forced expiratory volume in the first 6 s ratio. PEF%: peak expiratory flow in predicted. FEF25–75%: forced expiratory flow between 25–75% in predicted. FET: forced expiratory time. FENO: fractional exhaled nitric oxide. Model 1 was adjusted for age and gender. Model 2 was adjusted for age, gender, race/ethnicity, education level, and poverty-to-income ratio. Model 3 was adjusted for age, gender, race/ethnicity, education level, poverty-to-income ratio, body mass index, alcohol, and cotinine.

## DISCUSSION

Total of 5121 participants were included from NHANES 2007–2012, we tried to elucidate the potential relationship between urinary NNAL and lung function. High levels of urinary NNAL were associated with poorer lung function compared to groups with low levels of urinary NNAL, as reflected in spirometry values. And urinary NNAL seemed to be associated stronger with spirometry values in males. Our findings substantiate the detrimental impact of smoking on respiratory health and underscore the imperative need for targeted smoking cessation interventions within high-risk demographic groups.

Many previous studies have shown that cigarette exposure could have a profound effect on spirometry values, similar to our findings. Cigarette exposure accelerated the decline in FEV1 among adults^16^. FEV1 and FEV1/FVC% were decreased in smokers compared to non-smokers, and early smoking cessation mitigates the trend of lung function decline^17^. However, exposure to secondhand smoke causes respiratory symptoms but does not affect lung function^18^. A recent descriptive study indicated a significant decline in FENO in smokers and suggested that FENO levels could be used as a marker of smoking intensity^19^. However, the effect of age on FENO should not be ignored^20^, and it was valuable in assessing the effect of smoking on FENO. FEV3/FEV6% has been proposed as an indicator of peripheral airway disease and reduced FEV3/FEV6% was very common in smokers^21^. FEV1/FEV6% was highly valuable in the diagnosis of obstructive ventilation disorders and predicts lung function decline in smokers, suggesting a higher risk of COPD ^22^. In patients with COPD, assessment of FEV1/FEV6% showed that it was also an independent risk factor for hospitalization and death^23^. Environmental tobacco smoke (ETS) exposure altered lung function with significant reductions in FEV1, FVC, and FEF25-75%^24^. In COPD patients with smoking, large artery intima-media thickness was negatively correlated with FEV1/FVC% and FEF25-75%^25^. Small airway dysfunction occurred in non-smokers, frequently exposed to secondhand smoke with lower FVC, FEV1/FCV1%, and PEF%^26^. In our study, the effect of urinary NNAL on spirometry values was consistent with smoking, demonstrating that urinary NNAL was a very promising indicator of cigarette exposure. An assessment of the levels of urinary NNAL could reflect the respiratory health of the individual.

Interestingly, urinary NNAL was positively correlated with FET in our study. In general, with the preservation of lung emptying capacity, FET was prolonged in patients with obstructive lung disease due to reduced FEV1^27^. Previous study showed that FET increased in smoking populations and was associated with age and BMI^28^. In our study, higher urinary NNAL levels had higher FEV1, which might be responsible for the longer FET. The exact mechanisms explaining the relationship between urinary NNAL and lung function might be very complex and require further basic research to demonstrate. Chronic smoking can lead to altered lung ventilation and impaired airflow exchange^29^. Smoking caused chronic inflammation, an increase in white blood cells and red blood cells, and had a long-term effect on white blood cells^30^. These factors might contribute to the altered ventilation of the lungs. Smoking exposure causes damage to lung tissue, which further leads to altered lung function.

There were gender differences in the relationship between urinary NNAL and lung function in our study. After adjusting for all covariates, all indicators were associated with urinary NNAL in males except FVC%. This was not the case in females. Estrogen has been reported to have a protective effect against chronic lung injury and lung fibrosis^31^. Basic research has also shown that female sex hormones also reduce acute lung inflammation^32^. However, few have specifically investigated this gender-induced difference. In conclusion, gender differences in the relationship between urinary NNAL and lung function need to be further elucidated.

### Strengths and limitations

Our study has several strengths. First, our study sample was representative of the entire United States and weighted, including multiple ethnic groups and providing strong evidence of the effects of cigarettes on humans, especially the respiratory system. Second, the urinary NNAL was an indicator detected in human urine, which is more accurate than verbal active reporting of smoking and uninformed secondhand smoke exposure (which is prone to response bias), and provided a more comprehensive response to tobacco exposure in the population. Third, we excluded people with respiratory disease and adjusted for covariates including sociodemographics, lifestyle, and BMI to reduce confounding. Finally, we also conducted subgroup analyses of gender to understand the effect between males and females on the relationship between the urinary NNAL and lung function, and to select key populations that would provide the basis for better advocacy for smoking cessation.

The limitation of our study was mainly that it was cross-sectional, which prevented us from drawing a causal and temporal relationship between urinary NNAL and lung function. In addition, assessing the relationship between urinary NNAL and lung function might require a longer period of exposure or follow-up. In addition, we did not have enough sample size to exclude people with some diseases that might affect lung function in the population we chose; many factors that might affect lung function cannot all be taken into account. A larger population might be needed to be investigated in the future. Finally, the relationship between urinary NNAL and lung function may also be necessary for studies in children and adolescents. In the future, prospective studies are needed, notably in long-term smokers and those exposed to secondhand smoke. However, the relationship between the duration of cessation and lung function should also be included in those who quit smoking.

## CONCLUSIONS

Our cross-sectional investigation revealed that NNAL, a robust biomarker indicative of tobacco exposure, was associated with several spirometry parameters, including FEV1/FVC% and FEV1%, among others. Drawing from our study outcomes, we advocate for the urgency of smoking cessation, emphasizing the pivotal need to enhance smoking cessation efforts. We also underscore the importance of preventing non-smokers from any form of tobacco exposure. Furthermore, we envisage a future where urinary NNAL could be employed as a viable indicator within the techniques for evaluating lung health, potentially enhancing the precision of health assessments. Smoking cessation initiatives should thus retain a prominent place on the public health agenda.

## Data Availability

The data supporting this research are available from the following source: https://www.cdc.gov/nchs/nhanes/index.htm
